# Association between systemic inflammatory response index and bone turnover markers in Chinese patients with osteoporotic fractures: a retrospective cross-sectional study

**DOI:** 10.3389/fmed.2024.1404152

**Published:** 2024-07-11

**Authors:** Peng Zhou, Ke Lu, Chong Li, Min-zhe Xu, Yao-wei Ye, Hui-qiang Shan, Yi Yin

**Affiliations:** ^1^Department of Orthopedics, Gusu School, Nanjing Medical University, The First People's Hospital of Kunshan, Suzhou, Jiangsu, China; ^2^Department of Orthopedics, Affiliated Kunshan Hospital of Jiangsu University, Suzhou, Jiangsu, China

**Keywords:** systemic inflammatory response index, bone turnover markers, osteoporotic fractures, inflammation, osteoporosis

## Abstract

**Background:**

The systemic inflammatory response index (SIRI) is a novel composite biomarker of inflammation. However, there is limited information on its use in the context of osteoporotic fractures. Hence, this study aimed to investigate the association between baseline SIRI values and bone turnover markers (BTMs) in Chinese patients diagnosed with osteoporotic fractures (OPFs), to offer a more precise method for assessing bone health and inflammation in clinical settings.

**Methods:**

A retrospective cross-sectional study was conducted on 3,558 hospitalized patients with OPFs who required surgery or hospitalization at the First People’s Hospital of Kunshan City from January 2017 to July 2022. Baseline measurements of SIRI, β-CTX (beta-C-terminal telopeptide of type I collagen), and P1NP (procollagen type I N-terminal propeptide) were obtained. The analyses were adjusted for variables, including age, sex, body mass index (BMI), and other initial laboratory and clinical findings. Furthermore, multivariable logistic regression, smooth curve fitting, and threshold analysis were also performed.

**Results:**

The results revealed a negative correlation between baseline SIRI values and both β-CTX and P1NP levels. After adjusting for covariates in the regression analysis, each unit increase in SIRI was found to be inked to a reduction of 0.04 (*β* = −0.04; 95% confidence interval [CI], −0.05 to −0.03; with *p*-value <0.001) in β-CTX levels and a decrease of 3.77 (*β* = 3.77; 95% CI, 5.07 to 2.47; with *p*-value <0.001) in P1NP levels. Furthermore, a curvilinear relationship and threshold effect were also identified. Turning points were identified at SIRI values of 1.41 and 1.63 on the adjusted smooth curve.

**Conclusion:**

The results showed a negative correlation between the baseline SIRI value and β-CTX level, as well as the level of P1NP. This suggests a possible link between the systemic inflammatory response and reduced bone metabolism. If these findings are verified, SIRI has the potential to function as a predictive indicator for BTMs. Nevertheless, additional research is necessary to verify these findings.

## Introduction

Osteoporosis (OP) is a common disorder of bone metabolism defined by reduced bone mass and degradation of bone tissue (1), often leading to fragile and easily fractured bones (2), especially in women over the age of 55 and men over the age of 65. OP is diagnosed according to internationally recognized definitions when the bone density is >2.5 standard deviations below the mean of young healthy individuals (3). Most patients have secondary causes of OP, ranging from endocrine disorders to chronic inflammation and genetic diseases, which can contribute to the development of the disease (4). The prevalence rate of OP in the elderly population in China is approximately 39.4% (5). OP imposes a significant burden on both patients’ health and the economy (6, 7). It increases the risk of fractures, reducing the patients’ quality of life and increasing the likelihood of hospitalization, disability, and potentially even mortality (8). Global estimates indicate that an OP associated fracture occurs every three seconds, resulting in about 8.9 million instances each year (9).

Inflammation is known to promote bone resorption and is acknowledged as a risk factor for OP (10). Other factors, apart from inflammation, are also associated with the development of OP including age, sex, genetic factors, malnutrition, lack of exercise, smoking, marital status, and excessive alcohol consumption (11, 12). Early screening, diagnosis, and therapy are critical for the prevention and management of OP since they can decrease the likelihood of fractures and enhance patients’ quality of life (13).

Bone turnover markers (BTMs) are biochemical indicators that reflect the dynamic interplay between bone formation and resorption and can be measured in the serum, plasma, or urine (14, 15). In the present study, procollagen type 1 N-terminal propeptide (P1NP), and beta-C-terminal telopeptide of type I collagen (β-CTX) were studied as representative BTMs. These are among the most important BTMs. P1NP is a crucial constituent of the bone matrix and is released during the synthesis of type I collagen synthesis and its subsequent integration into the bone matrix, and thus qualifies as a reliable indicator of bone formation (16). P1NP levels are correlated with osteoblast activity (17).

β-CTX is a peptide fragment of collagen that is released into the bloodstream during the process of bone resorption and thus serves as an indicator of bone metabolism (18). The link between β-CTX and OP is well-established (19). Both β-CTX and P1NP are used routinely to monitor OP or predict the prognosis of individuals with disorders of bone metabolism.

The systemic inflammatory response index (SIRI) is a newly developed biomarker for inflammation that takes into account the absolute numbers of neutrophils, monocytes, and lymphocytes. The SIRI indicates the magnitude of the body’s inflammatory reaction (20). Previous studies have reported its extensive application in various cardiovascular diseases including stroke (21), ischemic heart disease (22), acute coronary syndrome (20), and aortic dissection (23). In addition, the SIRI has been employed in studies involving cervical cancer (24), COVID-19 (25), and ankylosing spondylitis (26). However, the correlation between the SIRI and OP, particularly with BTMs, is relatively unexplored with minimal data. To fill this gap, the present study aimd to determine the relation between the baseline SIRI and BTM levels (β-CTX and P1NP) in individuals with osteoporotic fractures (OPFs) to fill this research gap.

## Materials and methods

### Ethical statement

The study received approval from the Ethics Committee of the Affiliated Kunshan Hospital of Jiangsu University, Suzhou, China (approval No. 2021–06-015-K01), and adhered to the principles outlined in the Declaration of Helsinki. The patients’ identities were concealed to ensure an unbiased investigation. All patients provided written informed consent.

### Study design and patient clinical cohorts

In the present study, a retrospective cross-sectional analysis was performed using patient data collected from January 2017 to July 2022. The medical information of the patients was obtained from Kunshan Hospital, affiliated with Jiangsu University, Suzhou, China. The study involved a cohort of 3,558 patients with OPF who underwent surgical procedures or required hospitalization. All participants received blood tests while they were hospitalized. The diagnosis of OP was established based on the following criteria: (1) The existence of bone instability and fractures without any accompanying metabolic bone disorders, coupled with a standard bone mineral density (BMD) (T-score); (2) verification of osteoporosis (OP) using a T-score of −2.5 or below, even in the absence of a prevailing bone fracture (27). The exclusion criteria were as follows: (1) Missing or incomplete records; (2) Multiple or pathological hip fractures; (3) Diagnosed with other diseases that interfere with bone metabolism (such as thyroid diseases, parathyroid-related diseases, diabetes, gonadal diseases); (4) Presence of an autoimmune disease such as systemic lupus erythematosus; (5) Long-term use of drugs that affect bone metabolism; (6) SIRI>15 (28).

### Exposure and outcome variables

Levels of SIRI, β-CTX, and P1NP were assessed in a cohort of 732 individuals. The complete blood counts of the patients were determined and the SIRI was computed as the exposure variable. Neutrophils, monocytes, and lymphocytes were measured using flow cytometry with nuclear staining on the Sysmex XN-10 (B4) hematology analyzer. The outcome variables investigated were the levels of β-CTX and P1NP. The β-CTX and P1NP levels in patients were measured using automated electrochemiluminescence immunoassays (ECLIA) from Roche Diagnostics in (Mannheim, Germany). All measurements were collected using the same instrument and the same experienced operator following standardized protocols.

### Covariate variables

The covariate variables including age, sex, body mass index (BMI), high-density lipoprotein (HDL), total cholesterol (TC), hypertension, diabetes, calcium, platelet count, smoking status, alcohol consumption, and the Charlson comorbidity index (CCI) (29) were measured and recorded. Calcium levels were measured using a Beckman AU5800 automated biochemistry analyzer, employing the Arsenazo III method. Platelet counts were determined by flow cytometry with impedance on a Sysmex XN-10 (B4) hematology analyzer, while HDL was measured on the Beckman AU5800 automated biochemistry analyzer employing the direct approach. All clinical indicators were assessed within three days of admission.

### Statistical analyses

The data related to demographics, laboratory tests, and clinical outcomes are presented as either the median with the interquartile range (the 25th and 75th percentiles) or the mean ± standard deviation (SD). The data are presented in the form of frequencies (expressed as percentages) for each category. Categorical data were analyzed using either Pearson’s chi-square test or Fisher’s exact test for univariate analysis. Independent-sample tests were used to compare normally distributed continuous data, while the Mann–Whitney U test was used for non-normally distributed continuous data. The association between the attributes of OPFs and the BTMs, β-CTX, and P1NP, was also investigated using univariate analysis.

### Model construction

The Generalized Estimating Equation (GEE) and Generalized Additive Model (GAM) are two common statistical modeling approaches. GEE models the average response and correlations by specifying a working correlation structure, suitable for handling correlated data such as longitudinal or clustered data. In contrast, the GAM employs flexible nonparametric smoothing functions to explore complex nonlinear relationships between the response and predictors, without assuming parametric forms. Both require specifying the response distribution, formulating the mean model, and using iterative algorithms to estimate parameters. Researchers can then evaluate model fit and perform statistical inference.

The GEE was employed for appropriate adjustment of covariates and investigation of the independent relationship between SIRI levels and β-CTX and P1NP in OPFs. The models that were developed included unadjusted and slightly adjusted models, referred to as Model 1 and Model 2, respectively, as well as the fully adjusted model, termed Model 3. Firstly, a variance inflation factor (VIF) analysis was conducted to detect any collinearity among the covariates. Subsequently, decisions were taken to modify these elements based on the following criteria: (1) A modification in the matched odds ratio (OR) by ≥10% was observed upon the addition or removal of covariates in the basic or full model, respectively; (2) Variables that satisfied criterion 1 or had a *p*-value less than 0.1 in the univariate model (30). Model 3 employed both criteria 1 and 2 to adjust for covariates. This resulted in the development of three models, namely, Model 1, which was left unadjusted, and Model 2 (minimally adjusted model), which included covariate adjustments for age, sex, BMI, smoking status, alcohol consumption, hypertension, and diabetes CCI levels, and Model 3, which additionally included covariates such as calcium, HDL, total cholesterol, and the platelet count.

The GAM was used to detect possible non-linear associations. After the identification of these correlations, a two-piecewise linear regression model was used to determine the threshold effects in the resulting smoothing curves. A recursive approach was used to independently determine the inflection point, employing a maximum-likelihood model when the curves exhibited a clear ratio (31). The robustness of the studies and changes among patient subgroups were assessed by conducting subgroup analyses, stratifying patients based on specific covariates. Subgroup interactions and modifications were analyzed using the likelihood ratio test (LRT).

The R packages from The R Foundation^1^ and Empower Stats from X&Y Solutions, Inc., MA, USA^2^ were utilized for all analyses. A significance criterion of *p* < 0.05, using a two-tailed test, was employed.

## Results

### Clinical and demographic traits of subjects

**Figure 1:**
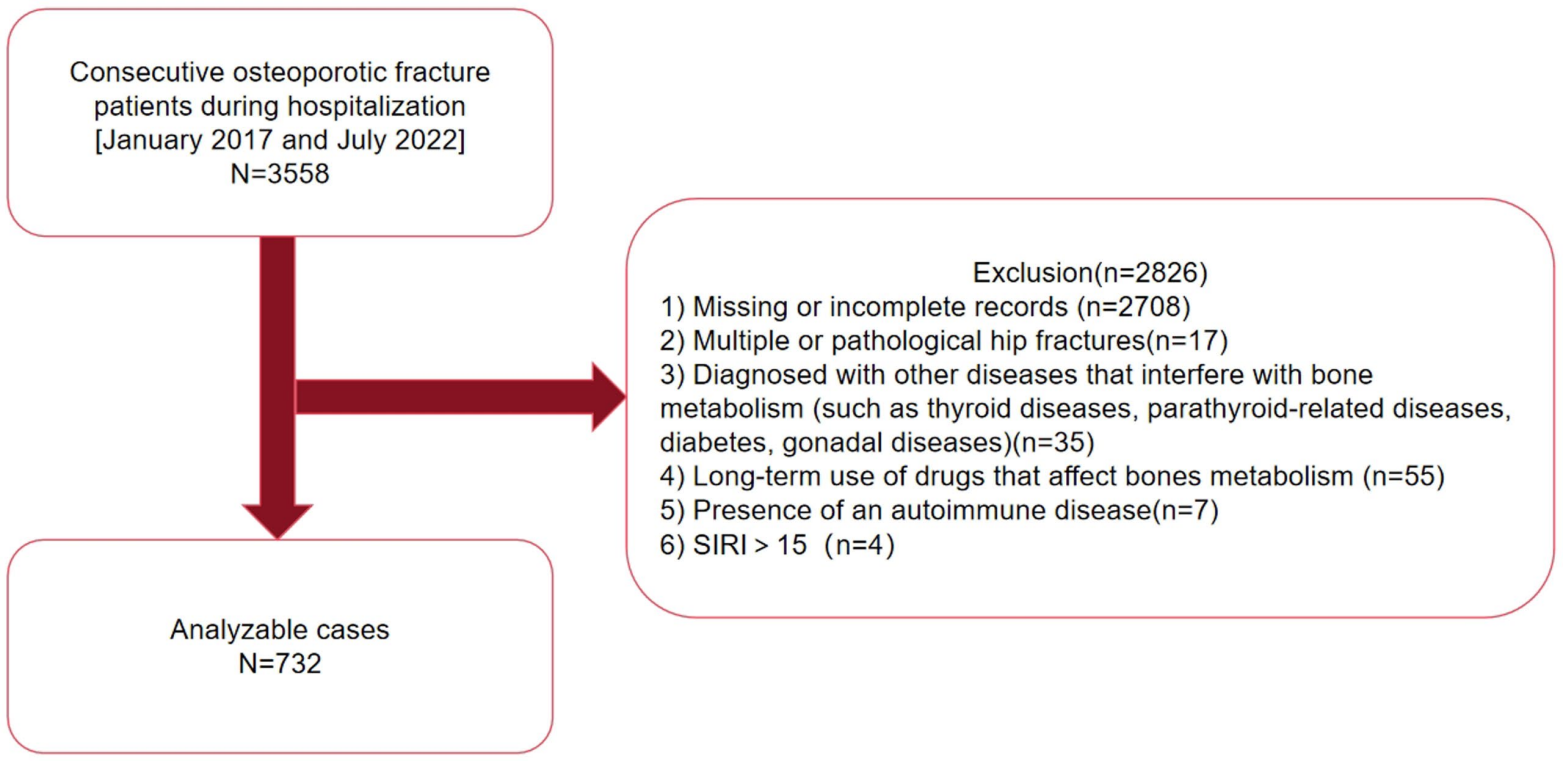
A schematic representation of the study design.

According to the eligibility criteria depicted in Figure 1, a total of 732 patients were treated between January 1, 2017, to July 27, 2022, were ultimately included in the analysis. Table 1 summarizes the baseline characteristics of the hospitalized patients (*n* = 732), of whom 33.61% were male and 66.39% female, with a mean age of 69.04 ± 11.02 years.

**Table 1:** Characteristics of study participants.

Characteristics	Mean (SD) Median (Q1-Q3)^a^
Age, years	69.04 (11.02) 68.00 (60.00–77.00)
BMI, kg/m^2^	22.96 (3.35) 23.13 (20.70–25.10)
TC, mmol/L	4.35 (0.89) 4.34 (3.71–4.92)
HDL, mmol/L	1.33 (0.29) 1.30 (1.14–1.50)
Calcium, mmol/L	2.22 (0.12) 2.22 (2.14–2.31)
Platelet count, ×10^9^/L	177.12 (61.18) 169.00 (137.00–205.00)
β-CTX, ng/mL	0.53 (0.28) 0.48 (0.33–0.69)
P1NP, ug/L	57.72 (30.72) 51.00 (38.00–69.00)
SIRI	2.40 (2.18) 1.73 (0.98–2.98)
*N* (%)	
Sex, *N* (%)	
female	486 (66.39%)
male	246 (33.61%)
Smoke, *N* (%)	
No	684 (93.44%)
Yes	48 (6.56%)
Drink, *N* (%)	
No	704 (96.17%)
Yes	28 (3.83%)
Hypertension, *N* (%)	
No	642 (87.70%)
Yes	90 (12.30%)
Diabetes, *N* (%)	
No	702 (95.90%)
Yes	30 (4.10%)
Age, years, *N* (%)	
>50, ≤70	420 (57.38%)
>70	312 (42.62%)
CCI, *N* (%)	
0	655 (89.48%)
1	58 (7.92%)
≥2	19 (2.60%)
BMI, kg/m^2^, *N* (%)	
≤18.5	73 (9.97%)
>18.5, ≤23.9	362 (49.45%)
>23.9, ≤28.0	255 (34.84%)
>28	42 (5.74%)

^a^For continuous variables.SD, standard deviation; Q1, first quartile; Q3, third quartile; CI, confidence interval; BMI, body mass index; TC, total cholesterol; HDL, high-density lipoprotein; β-CTX, beta-C-terminal telopeptide of type I collagen; P1NP, procollagen type I N-terminal propeptide; SIRI, systemic inflammatory response index; CCI, Charlson comorbidity index.

The subjects in this study exhibited a mean Systemic Immune-Inflammation Index (SIRI) value of 2.40 ± 2.18, a mean β-CTX value of 0.53 ± 0.28 ng/mL, and a mean P1N*p* value of 57.72 ± 30.72 μg/L. Observations were conducted to identify variations in the co-variate variables among the patient cohort.

### Univariate analysis of BTMs

A univariate analysis was performed to investigate the relation between β-CTX and P1NP with covariate variables (Table 2). No significant relationships were found between the investigated variables and β-CTX or P1NP in the patients with OPF.

**Table 2:** Univariate analysis for bone metabolism markers.

Characteristics	Statistics^a^	β-CTX β(95%CI) *p*-value	P1NP β(95%CI) *p*-value
Age, years	69.04 ± 11.02	0.00 (−0.00, 0.00) 0.35	−0.02 (−0.22, 0.19) 0.88
BMI, kg/m^2^	22.96 ± 3.35	0.00 (−0.01, 0.01) 0.73	0.09 (−0.57, 0.76) 0.79
TC, mmol/L	4.35 ± 0.89	−0.03 (−0.05, −0.00) 0.04	−2.61 (−5.57, 0.35) 0.09
HDL, mmol/L	1.33 ± 0.29	−0.12 (−0.20, −0.04) 0.004	−8.57 (−17.61, 0.46) 0.06
Calcium, mmol/L	2.22 ± 0.12	0.18 (0.01, 0.34) 0.04	15.96 (−2.11, 34.03) 0.08
Platelet count, ×10^9^/L	177.12 ± 61.18	0.00 (0.00, 0.00) 0.003	0.05 (0.01, 0.08) 0.01
SIRI	2.40 ± 2.18	−0.03 (−0.04, −0.02) <0.001	−2.70 (−3.70, −1.70) <0.001
Sex, *N* (%)			
female	486 (66.39%)	Reference	Reference
male	246 (33.61%)	−0.01 (−0.05, 0.04) 0.72	1.13 (−3.59, 5.84) 0.64
Smoke, *N* (%)			
No	684 (93.44%)	Reference	Reference
Yes	48 (6.56%)	−0.07 (−0.15, 0.01) 0.11	−4.43 (−13.42, 4.56) 0.33
Drink, *N* (%)			
No	704 (96.17%)	Reference	Reference
Yes	28 (3.83%)	−0.04 (−0.14, 0.07) 0.51	−3.95 (−15.55, 7.66) 0.51
Hypertension, *N* (%)			
no	642 (87.70%)	Reference	Reference
yes	90 (12.30%)	0.05 (−0.01, 0.11) 0.12	5.00 (−1.77, 11.77) 0.15
Diabetes, *N* (%)			
No	702 (95.90%)	Reference	Reference
Yes	30 (4.10%)	0.01 (−0.09, 0.11) 0.86	3.62 (−7.61, 14.86) 0.53
Age, years, *N* (%)			
>50, ≤70	420 (57.38%)	Reference	Reference
>70	312 (42.62%)	0.01 (−0.03, 0.05) 0.67	−1.68 (−6.18, 2.82) 0.46
CCI, *N* (%)			
0	655 (89.48%)	Reference	Reference
1	58 (7.92%)	−0.01 (−0.08, 0.07) 0.82	2.76 (−5.50, 11.02) 0.51
≥2	19 (2.60%)	−0.01 (−0.14, 0.11) 0.83	−0.36 (−14.38, 13.67) 0.96
BMI, kg/m^2^, *N* (%)			
≤18.5	73 (9.97%)	Reference	Reference
>18.5, ≤23.9	362 (49.45%)	0.03 (−0.04, 0.10) 0.34	6.33 (−1.39, 14.04) 0.11
>23.9, ≤28.0	255 (34.84%)	−0.01 (−0.08, 0.06) 0.83	1.43 (−6.55, 9.41) 0.73
>28.0	42 (5.74%)	0.06 (−0.05, 0.16) 0.30	5.05 (−6.60, 16.69) 0.40

^a^For continuous variables.^b^The dependent variable was bone turnover markers and β is the result of univariate analysis for bone turnover markers.BMI, body mass index; TC, total cholesterol; HDL, high-density lipoprotein; β-CTX, beta-C-terminal telopeptide of type I collagen; P1NP, procollagen type I N-terminal propeptide; SIRI, systemic inflammatory response index; CCI, Charlson comorbidity index.

### Analysis of the relationship between SIRI and BTMs

Three models were used in the subsequent phase to analyze the correlation between SIRI and both BTMs (β-CTX and P1NP) in participants with OP (Table 3). In the unadjusted Model 1, there was a significant correlation between SIRI and β-CTX (*β* = −0.03, 95% CI: −0.04 to −0.02, *p* < 0.001) (β is the regression coefficient in the linear regression model, representing the magnitude and direction of the association between SIRI and BTMs). Similarly, a significant correlation was found between SIRI and P1NP (*β* = −2.87, 95% CI: −3.70 to −1.70, *p* < 0.001). After adjusting for variables including age, sex, BMI, smoking status, alcohol intake, CCI, hypertension, and diabetes in Model 2, the observed relationships were consistent. The SIRI remained significantly associated with both β-CTX (*β* = −0.03, 95% CI: −0.04 to −0.02, *p* < 0.001) and P1NP (*β* = −2.83, 95% CI: −4.02 to −1.64, *p* < 0.001). Expanding on Model 2, Model 3 included further adjustments for HDL, TC, calcium, and platelet count, and consistently showed a negative correlation. The SIRI remained significantly associated with β-CTX (*β* = −0.04, 95% CI: −0.05 to −0.03, *p* < 0.001) and P1NP (*β* = −3.77, 95% CI: −5.07 to −2.47, *p* < 0.001).

**Table 3:** Association between SIRI and bone turnover markers in different models.

	Model 1^a^ *N* = 732 β (95%CI) *P*-value	Model 2^b^ *N* = 732 β (95%CI) *P*-value	Model 3^c^ *N* = 574 β (95%CI) *P*-value
β-CTX	−0.03 (−0.04, −0.02) <0.001	−0.03 (−0.04, −0.02) <0.001	−0.04 (−0.05, −0.03) <0.001
P1NP	−2.70 (−3.70, −1.70) <0.001	−2.83 (−4.02, −1.64) <0.001	−3.77 (−5.07, −2.47) <0.001

^a^No adjustment.^b^Adjusted for age, sex, BMI, smoke, drink, CCI, hypertension, diabetes.^c^Adjusted for age, sex, BMI, smoke, drink, CCI, hypertension, diabetes, HDL, TC, calcium; platelet count. BMI, body mass index; TC, total cholesterol; HDL, high-density lipoprotein; β-CTX, beta-C-terminal telopeptide of type I collagen; P1NP, procollagen type I N-terminal propeptide; SIRI, systemic inflammatory response index; CCI, Charlson comorbidity index.

Additional subgroup analysis was conducted to assess the robustness of Model 3 by categorizing patients with OPF according to various characteristics such as age, sex, BMI, smoking status, alcohol consumption, CCI, hypertension, diabetes, HDL, TC, calcium, and platelet count. Adjustment was established for those covariates that were not utilized for stratification. The studies showed consistent patterns in the results, with no detected interactions due to stratification (all *p* < 0.05, Table 4).

**Table 4:** Subgroup analysis between SIRI and bone turnover markers.

	*N*	β-CTX^a^	P1NP^a^
		β (95% CI) *p*-value	β (95% CI) *p*-value
Age, years, *N* (%)			
>50, ≤70	420	−0.04 (−0.05, −0.02) <0.001	−3.53 (−5.27, −1.78) <0.001
>70	312	−0.04 (−0.06, −0.02) <0.001	−4.47 (−6.43, −2.51) <0.001
Sex, *N* (%)			
Female	486	Reference	Reference
Male	246	−0.03 (−0.05, 0.01) 0.007	−3.59 (−5.94, −1.24) 0.003
BMI, kg/m^2^, *N* (%)			
≤18.5	73	−0.03 (−0.05, −0.01) 0.004	−2.50 (−4.88, −0.12) 0.05
>18.5, ≤23.9	362	−0.04 (−0.06, −0.02) <0.001	−4.28 (−6.54, −2.03) <0.001
>23.9	297	−0.04 (−0.06, −0.02) <0.001	−3.45 (−5.49, −1.40) 0.001
Smoke, *N* (%)			
No	684	−0.04 (−0.05, −0.03) <0.001	−3.88 (−5.17, −2.60) <0.001
Yes	48	Reference	Reference
Drink, *N* (%)			
No	704	−0.04 (−0.05, −0.03) <0.001	−3.76 (−5.12, −2.39) <0.001
Yes	28	Reference	Reference
Hypertension, *N* (%)			
No	642	−0.03 (−0.05, −0.02) <0.001	−3.48 (−4.81, −2.14) <0.001
Yes	90	−0.08 (−0.12, −0.04) <0.001	−5.04 (−10.27, 0.19) 0.06
Diabetes, *N* (%)			
No	702	−0.04(−0.05, −0.03) <0.001	−3.68 (−5.12, −2.46) <0.001
Yes	30	Reference	Reference
CCI, *N* (%)			
0	655	Reference	Reference
≥1	77	−0.05 (−0.07, −0.02) 0.002	−4.50 (−7.20, −1.80) 0.002
TC, mmol/L			
Low	249	−0.04 (−0.05, −0.02) <0.001	−3.12 (−5.35, −0.90) 0.006
High	325	−0.04 (−0.05, −0.02) <0.001	−4.10 (−5.67, −2.54) <0.001
HDL, mmol/L			
Low	292	−0.04 (−0.06, −0.03) <0.001	−3.73 (−5.92, −1.55) 0.001
High	282	−0.03 (−0.05, −0.02) <0.001	−0.04 (−3.27, −1.66) <0.001
Calcium, mmol/L			
Low	334	−0.03 (−0.05, −0.02) <0.001	−3.10 (−5.34, −0.86) 0.007
High	395	−0.04 (−0.05, −0.02) <0.001	−3.93 (−5.52, −2.34) <0.001
Platelet count, ×10^9^/L			
Low	367	−0.04 (−0.06, −0.03) <0.001	−3.82 (−5.95, −1.70) <0.001
High	365	−0.03 (−0.05, −0.02) <0.001	−3.46 (−5.09, −1.84) <0.001

^a^Patients were stratified based on age, sex, BMI, smoking status, alcohol consumption, CCI, hypertension, diabetes, HDL, TC, calcium, and platelet count, and additional covariates not included in the stratification were adjusted for in the analysis. BMI, body mass index; TC, total cholesterol; HDL, high-density lipoprotein; β-CTX, beta-C-terminal telopeptide of type I collagen; P1NP, procollagen type I N-terminal propeptide; SIRI, systemic inflammatory response index; CCI, Charlson comorbidity index.

### Spline smoothing plot and threshold analysis

**Figure 2:**
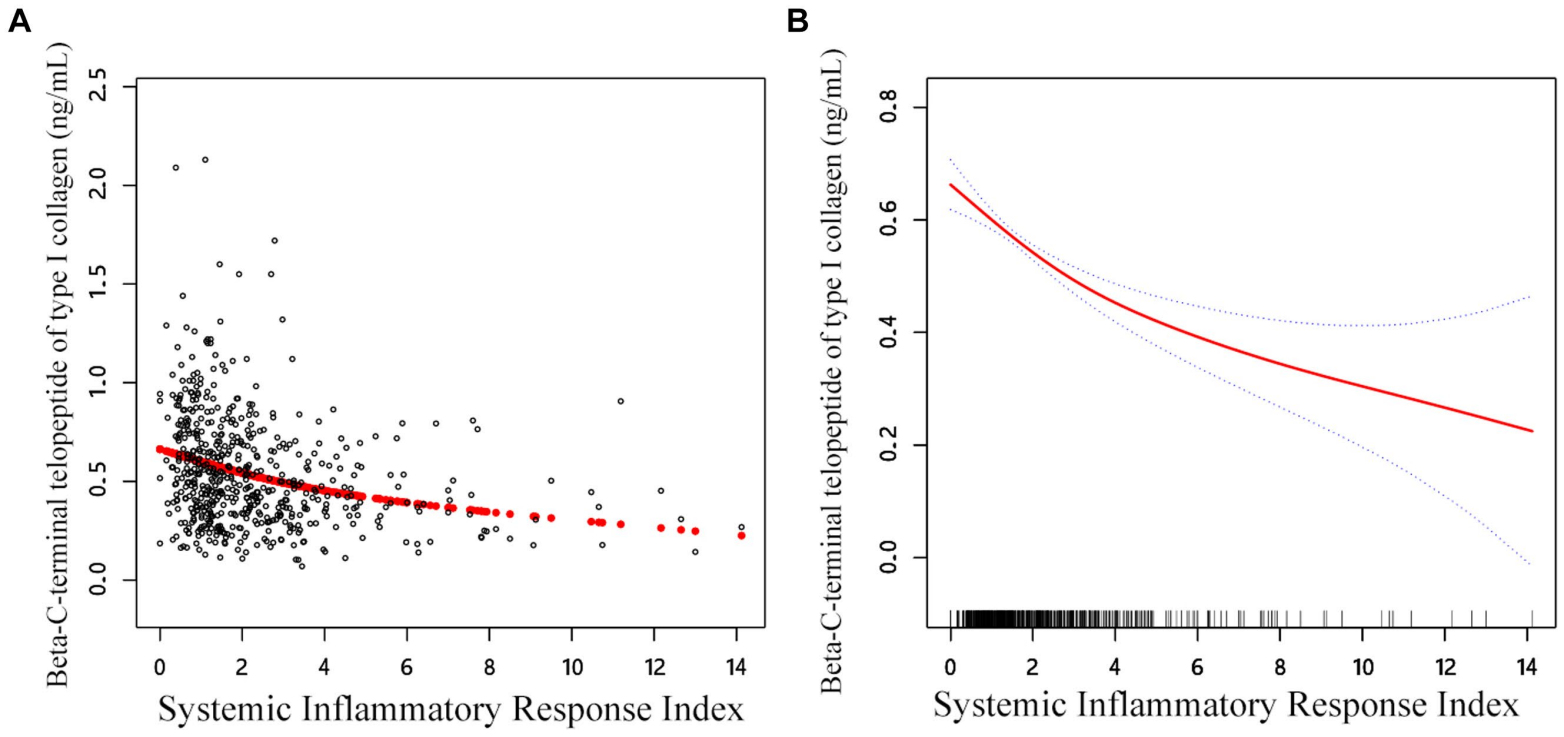
Adjusted smoothed curve analysis revealing the interplay between SIRI and β-CTX: **(A)** Each black point represents a single participant sample. **(B)** Solid red line represents the smooth curve fit between variables. Blue bands represent the 95% of confidence interval from the fit. Age, sex, BMI, smoke, drink, CCI, hypertension, diabetes, HDL, TC, calcium, and platelet count were adjusted.

The link between SIRI β-CTX and P1NP was then evaluated using graphical techniques to determine if it was linear or nonlinear (Figure 2). The GAM estimation revealed that, after accounting for covariate variables, there were distinct nonlinear relationships between SIRI and BTMs in the OPF population in the study. These associations were modeled using segmented linear regression, with the identified breakpoints (K-values) being 1.41 and 1.63, respectively (Table 5). To the left of the thresholds, there was a stronger negative correlation between SIRI and β-CTX, as well as with P1NP.

**Table 5:** Threshold analyses examining the relationship between SIRI and bone turnover markers.

	Model 3^a^	
	β-CTX	P1NP
Model A^b^		
One line slope	−0.04 (−0.05, −0.03) <0.001	−3.77 (−5.07, −2.47) <0.001
Model B^c^		
SIRI turning point (K)	1.41	1.63
<K	−0.13 (−0.20, −0.06) <0.001	−13.64 (−20.17, −7.11) <0.001
>K	−0.03 (−0.04, 0.02) <0.001	−2.35 (−3.94, −0.77) 0.004
Slope 2-Slope 1	0.01 (0.03, 0.18) 0.008	11.29 (3.96, 18.62) 0.003
LRT^d^	0.007	0.002

^a^Adjusted for age, sex, BMI, smoke, drink, CCI, hypertension, diabetes, HDL, TC, calcium, platelet count.^b^Linear analysis, *p* value < 0.05 indicates a linear relationship.^c^Nonlinear analysis.^d^*p* value < 0.05 means Model B is significantly different from Model A, which indicates a nonlinear relationship.BMI, body mass index; TC, total cholesterol; HDL, high-density lipoprotein; β-CTX, beta-C-terminal telopeptide of type I collagen; P1NP, procollagen type I N-terminal propeptide; SIRI, systemic inflammatory response index; CCI, Charlson comorbidity index.

**Figure 3:**
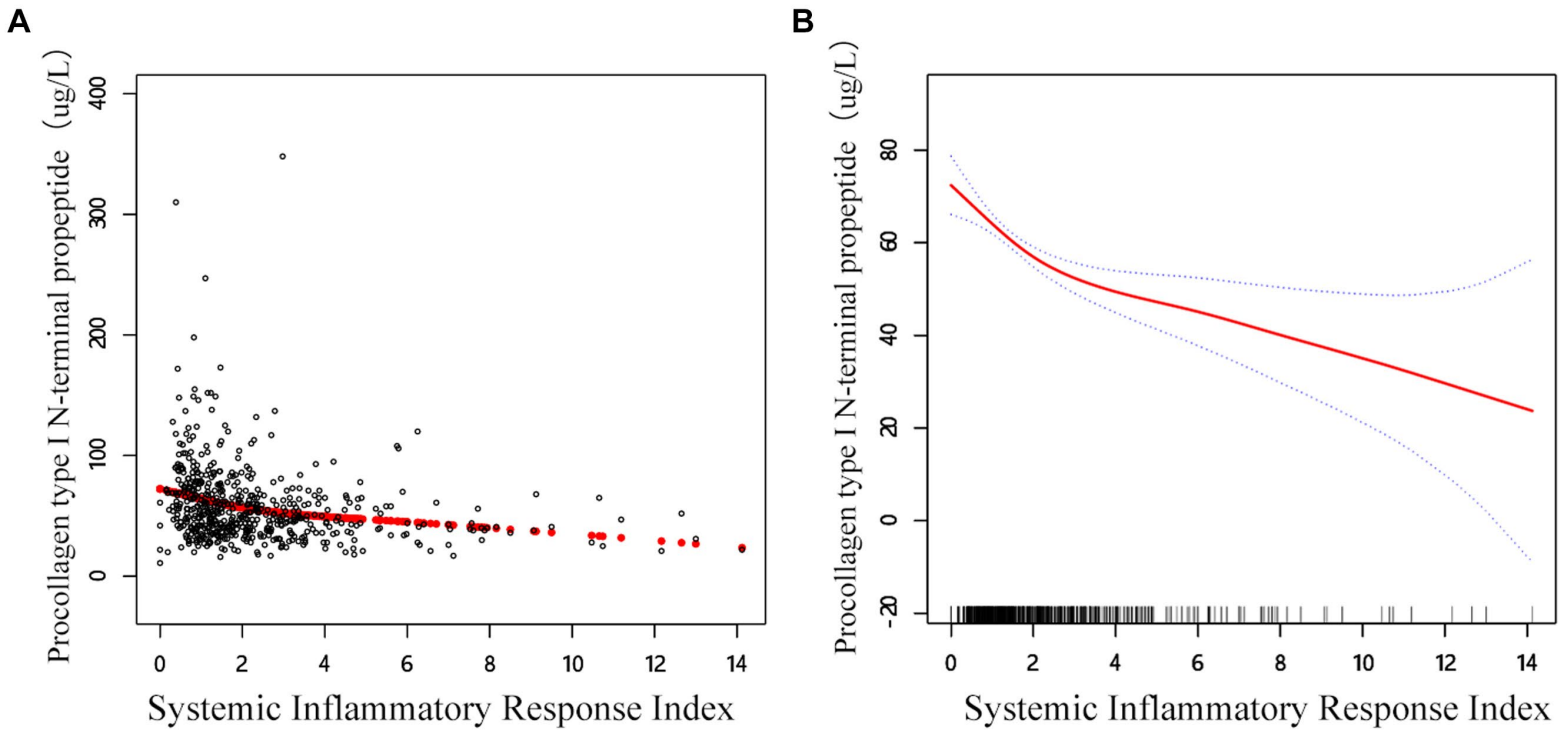
Adjusted smoothed curve analysis revealing the interplay between SIRI and P1NP: **(A)** Each black point represents a single participant sample. **(B)** Solid red line represents the smooth curve fit between variables. Blue bands represent the 95% of confidence interval from the fit. Age, sex, BMI, smoke, drink, CCI, hypertension, diabetes, HDL, TC, calcium, and platelet count are adjusted.

In the threshold studies for SIRI and β-CTX, the effect size on the left side of the threshold was −0.13 (95% CI: −0.20 to −0.06, *p* = 0.002). The effect size on the right side of the threshold was −0.03 (95% CI: −0.04 to −0.02, *p* < 0.001). Regarding the SIRI and P1NP thresholds, the effect size on the left side of the threshold was −13.64 (95% CI: −20.17 to −7.11, *p* < 0.001). The effect size on the right side of the threshold was −2.35 (95% CI: −3.94 to −0.77, *p* = 0.003) (Figure 3).

## Discussion

This cross-sectional study aimed to investigate the association between the SIRI and BTMs, including β-CTX and P1NP, in 3,558 patients hospitalized for osteoporotic fractures that required surgical intervention. The findings suggest that increased inflammation is associated with decreased bone metabolism.

The association between inflammation and OP has recently received attention, as the pivotal role of inflammation in the pathogenesis of OP is well-recognized. Clinical observations have indicated that systemic OP often co-exists with systemic periodic inflammation, while localized OP tends to be associated with regional inflammatory processes (32). Normal bone metabolism in the human body is dependent on achieving an equilibrium between bone resorption and formation (33). It is reported that various pro-inflammatory cytokines, including, IL-1, IL-6, TNF-α (34), and CRP, may cause persistent systemic and subclinical inflammation (35, 36). In response to these inflammatory stimuli, blood cells, especially neutrophils, are activated and recruited to the site (37), while other immune cells assume residence in the bone marrow (38). The SIRI value indicates alterations in blood parameters, indicative of a disturbance in the steady state of bone metabolism and a subsequent decline in the bone metabolism level (39). Inflammatory mechanisms underlying OP have also been proposed in recent years (40, 41). It is evident that inflammation alters bone homeostasis, resulting in reduced bone mass, weaker bone strength, and decreased bone density, as well as ultimately a decrease in bone turnover, all of which contribute to adverse outcomes in OPFs (42, 43). Increased circulating levels of pro-inflammatory cytokines and immune cells directly or indirectly affect bone turnover through various pathways (44, 45). To date, several studies have developed delivery systems using extracellular vesicles (EVs) secreted by mesenchymal stem cells (MSCs), targeting both the suppression of bone resorption and the promotion of bone formation and angiogenesis (46). This suggests the potential of anti-inflammatory/immune-based therapies for the treatment of OP.

The SIRI is a promising inflammatory marker that provides a comprehensive reflection of the body’s immune and inflammatory status (47). However, to the best of our knowledge, there is still insufficient information and evidence on the association between the SIRI and bone metabolism. Therefore, the objectives of the present study were to investigate the correlation between the SIRI and BTMs in patients with OPF to assess the predictive value of the SIRI in OPFs. We demonstrated a significant negative correlation between SIRI and BTMs (β-CTX and P1NP), indicating that increased systemic inflammation is associated with reduced bone metabolism, which typically suggests a poorer prognosis. These findings also suggest that SIRI values may serve as a tool for evaluating the risk of OPFs. Consistent with prior research (42, 48, 49), the present study demonstrated that a strong inflammatory response is detrimental to the prognosis of individuals with OP.

Notable advantages of this study encompass a nationally representative population and statistical models that adjusted for various important confounding factors. It is believed that these research results may apply to the general population. However, the study is subject to several limitations. First, the study used a cross-sectional design, which is only able to establish associations rather than demonstrate the temporal relationship between the SIRI and changes in BTMs. Furthermore, bone fractures can elicit acute-phase responses, leading to alterations in blood parameters. The study was unable to exclude the potential influence of fractures on inflammatory biomarkers, and thus represents a limitation of the research. The pathogenesis of OP is influenced by both hereditary and non-genetic factors. However, this data analysis largely focused on controlling for specific demographic and lifestyle variables. Further prospective research is required for a comprehensive understanding of the relevant linkages. In addition, the sample size was relatively small, consisting of only 732 subjects that could be analyzed. This calls for more extensive research in the future, involving detailed studies on real patient populations with different diseases.

## Conclusion

In conclusion, the finding of the study established a significant negative relationship between the SIRI and BTMs (β-CTX and P1NP) in individuals with OPFs. The results imply that increased inflammation is linked to reduced bone metabolism, often indicative of an unfavorable prognosis. These findings underscore the harmful impact of inflammation in OP and suggest that the assessment of SIRI values could serve as a valuable tool for evaluating the risk and prognosis of OPFs. Further research is required to unravel the underlying relationship between inflammation and OP and to validate the predictive utility of the SIRI in more extensive and diverse patient cohorts.

## Data availability statement

The original contributions presented in the study are included in the article/Supplementary material, further inquiries can be directed to the corresponding author.

## Ethics statement

The studies involving humans were approved by the IRB of Affiliated Kunshan Hospital of Jiangsu University. The studies were conducted in accordance with the local legislation and institutional requirements. The participants provided their written informed consent to participate in this study. Written informed consent was obtained from the individual(s) for the publication of any potentially identifiable images or data included in this article.

## Author contributions

PZ: Data curation, Formal analysis, Investigation, Resources, Software, Writing – original draft, Writing – review & editing. KL: Data curation, Methodology, Resources, Software, Supervision, Validation, Writing – review & editing. CL: Resources, Software, Supervision, Validation, Writing – review & editing. M-zX: Data curation, Resources, Software, Supervision, Validation, Writing – original draft. Y-wY: Investigation, Methodology, Resources, Supervision, Validation, Writing – original draft. H-qS: Resources, Software, Supervision, Validation, Writing – review & editing. YY: Supervision, Validation, Writing – review & editing.

## Footnotes

^1^ http://www.R-project.org

^2^ http://www.empowerstats.com
